# Flavour processing in semantic dementia

**DOI:** 10.1016/j.cortex.2009.07.002

**Published:** 2010-06

**Authors:** Katherine E. Piwnica-Worms, Rohani Omar, Julia C. Hailstone, Jason D. Warren

**Affiliations:** a Washington University in St Louis, Missouri, USA; b Dementia Research Centre, Institute of Neurology, University College London, UK

**Keywords:** Flavour, Taste, Olfaction, Gustation, Eating behaviour, Semantic dementia

## Abstract

The cognitive mechanisms for the analysis of flavour information remain poorly understood. Patients with semantic dementia (SD) could potentially provide a window on these mechanisms; however, while abnormal eating behaviour and altered food preferences are common in SD, flavour processing has been little studied in this disorder. Here we undertook a detailed investigation of flavour processing in three patients at different stages of SD. One patient with a clinical syndrome of logopenic aphasia (LPA) was studied as a disease control, and six healthy control subjects also participated. Olfaction was assessed using the University of Pennsylvania Smell Identification Test and processing of flavours was assessed using a novel battery to assess flavour perception, flavour identification, and congruence and affective valence of flavour combinations. Patients with SD performed equivalently to healthy controls on the perceptual subtest, while their ability to identify flavours or to determine congruence of flavour combinations was impaired. Classification of flavours according to affective valence was comparable to healthy controls. In contrast, the patient with LPA exhibited a perceptual deficit with relatively preserved identification of flavours, but impaired ability to determine flavour congruence, which did not benefit from affective valence. Olfactory and flavour identification performance was correlated in both patients and controls. We propose that SD produces a true deficit of flavour knowledge (an associative agnosia), while other peri-Sylvian pathologies may lead to deficient flavour perception. Our findings are consistent with emerging evidence from healthy subjects for a cortical hierarchy for processing flavour information, instantiated in a brain network that includes the insula, anterior temporal lobes and orbitofrontal cortex. The findings suggest a potential mechanism for the development of food fads and other abnormal eating behaviours.

The cognitive mechanisms for the analysis of flavour information remain poorly understood. Semantic dementia (SD), a canonical focal subtype of frontotemporal lobar degeneration, could potentially provide a window on these mechanisms: patients typically present with impaired word comprehension and vocabulary, however this is generally accompanied by non-verbal semantic deficits (Hodges & Patterson, 2007) and this may include defects of chemosensory knowledge (Rami et al., 2007; Luzzi et al., 2007). Typically there is focal atrophy of the left anterior and inferior temporal lobe (TL) on brain magnetic resonance imaging (MRI) at presentation, and involvement of the contralateral (right) anterior TL and orbitofrontal cortex (OFC) are common as SD evolves (Desgranges et al., 2007). It has been proposed that the core deficit in SD is multimodal impairment of brain knowledge systems, possibly implicating ‘amodal’ semantic processing mechanisms in the left anterior TL (Lambon Ralph and Patterson, 2008). While modalities other than verbal and visual knowledge have not been extensively investigated in SD, available evidence supports a multimodal breakdown of semantic processing, affecting visual objects, environmental sounds (Bozeat et al., 2000), odours (Rami et al., 2007; Luzzi et al., 2007) and tactile information (Coccia et al., 2004). However, there is little information about flavour processing in SD. This issue is of clinical as well as neurobiological relevance, as alterations in food preference, ‘food faddism’ or unusual food combinations are often salient in patients with SD (Snowden et al., 2001; Rosen et al., 2006). The brain basis for these behaviours–and in particular, any contribution from altered semantic processing of flavours–has not been fully defined, due in part to the challenges of assessing cortical olfactory and gustatory processing in the laboratory.

Elementary taste qualities (sweet, sour, bitter, salty, savoury) have only a limited relation to the rich diversity of flavours that characterise foods. Natural flavours are complex composites of gustatory, olfactory and other information derived from various sensory modalities, and comparatively little is known about the organisation of flavour knowledge in the healthy brain. In humans, the anterior insula contains primary gustatory cortex and pyriform cortex contains primary olfactory cortex, while the anterior TL and OFC are likely to contain higher-order gustatory and olfactory cortex (Savic, 2002; Rolls, 2005; Royet et al., 1999; Kareken et al., 2003; Small et al., 2001, 2004). These regions are likely to have specific roles processing different aspects of flavours, with representation of perceptual characteristics at earlier stages and semantic, evaluative and integrative processing leading to behavioural outputs at later stages of a cortical processing hierarchy. The limbic system, in particular the amygdala, is likely to be involved in evaluative processing of foods for factors such as pleasantness, intensity and novelty (Small et al., 2001; Hinton et al., 2004). Damage involving these areas has been shown to correlate with the development of relevant clinical deficits in SD and other degenerative pathologies: besides impaired identification of odours in SD (Rami et al., 2007; Luzzi et al., 2007), impaired identification of odours (Mendez and Ghajarnia, 2001; Rami et al., 2007) and flavours (Gorno-Tempini et al., 2004) has been described in the setting of FTLD with focal right temporal and inferior frontal lobe atrophy and in Alzheimer's disease (Broggio et al., 2001).

Here we set out to investigate flavour processing in SD using novel tests of flavour perception and knowledge. We hypothesised that patients with SD have an associative agnosia for flavours manifesting as impaired identification of flavours and abnormal processing of congruence in flavour combinations (defined here as the tendency of particular flavours to occur together in foods commonly consumed by individuals with a shared cultural background).

## 2 Methods

### 2.1 Subject details

Subject details are summarised in Table 1. Three patients (two male) with a clinical diagnosis of SD were studied (Cases 1–3). All fulfilled clinical consensus criteria for SD (Neary et al., 1998), with supportive neuropsychological and MRI features. Case 1 was assessed earlier in the course while Cases 2 and 3 had moderately severe disease based on estimated disease duration and brain MRI findings (Table 1). One male patient with a clinical diagnosis of the logopenic variant of primary progressive aphasia (LPA) was also studied (Case 4). The basis for LPA has not been fully defined, however it is neuropsychologically and radiologically distinct from SD (Rosen et al., 2006), and in a high proportion of cases is likely to represent a variant of Alzheimer's disease with focal atrophy of left peri-Sylvian cortex (Gorno-Tempini et al., 2008). Case 4 had a cerebrospinal fluid profile (raised level of total tau and depressed level of amyloid beta fraction) supportive of AD pathology. We included this case as a disease control for the SD cases, to assess the specificity of any flavour processing defect identified. Behavioural changes including abnormalities of eating behaviour were present in Cases 2 and 4. Patients had general neuropsychological profiles in keeping with their clinical diagnoses (Table 1): Cases 1–3 with SD had severe anomia and deficits of single word comprehension, with more variable impairments of non-verbal semantic (famous face) processing and episodic memory, while Case 4 with LPA had less severe anomia and verbal semantic impairment and well-preserved face recognition but impaired episodic memory and executive dysfunction.

Six healthy control subjects (four male) age-matched (mean 61.5 years; range 52–67 years) and of comparable educational and social background to the patients also participated. No subject had a history of significant head injury or other condition likely to affect peripheral gustatory or olfactory function and none was a smoker. All subjects gave their informed consent prior to their inclusion in the study, which was approved by the local Ethics Committee and performed in accordance with the ethical standards laid down in the Declaration of Helsinki.

### 2.2 Olfactory assessment

As retronasal olfactory processing contributes to processing of flavours in the mouth, all subjects completed the University of Pennsylvania Smell Identification Test (UPSIT), a 40 item 4-alternative-forced choice odour to word matching procedure (Doty et al., 1984). The UPSIT was designed to sample a wide range of common odorants, including both elementary and more complex odour classes; the foils used in the word arrays for each trial were designed to maximise olfactory perceptual distance between target and foil items, rather than semantic relatedness. While the UPSIT should not therefore be regarded as primarily a ‘semantic’ olfactory test of the kind developed specifically to assess olfactory recognition (Luzzi et al., 2007), we elected to use the UPSIT here because it has been normed and widely validated in older subjects and has been administered to patients with a variety of neurodegenerative conditions including SD (Rami et al., 2007). It therefore provides a robust background index of olfactory performance. Administration of the UPSIT was modified as previously described (Rami et al., 2007), such that both words and pictures were presented simultaneously for matching with each target odour: this was to reduce the potentially confounding effect of impaired word comprehension in the SD patients.

### 2.3 Experimental assessment of flavour processing

In order to characterise any deficit in the semantic processing of individual flavours or flavour combinations, we designed a novel battery incorporating tests of flavour perception, flavour identification and congruency of flavour combinations. Flavour stimuli were commercially available jelly bean candies (JellyBelly^®^). Jelly beans have been used previously to assess flavour processing in clinical settings (Gorno-Tempini et al., 2004), and offer the advantages of wide sampling from the flavour ‘space’ with relatively uniform stimulus quantity and presentation and minimal extraneous cues to flavour identity.

#### 2.3.1 Preliminary investigation

In a preliminary investigation, nine healthy British individuals (none of whom participated in the subsequent experiment) were presented with a list of 50 jelly bean flavours and asked to nominate usual (standard, congruent) and unusual (non-standard, incongruent) flavour combinations for British residents, based on their life experience of British cuisine. Flavour groupings spontaneously nominated by a majority of control participants (>5/9) were taken to represent ‘congruent’ flavour combinations; flavour groupings nominated by none of the participants were taken to represent ‘incongruent’ combinations.

#### 2.3.2 Flavour stimuli

Using the data generated from the initial pilot analysis, twenty target flavours were chosen and arranged to generate ten congruent (standard, or usual), and ten incongruent (non-standard, or unusual) flavour combinations (see Table 2). Whereas incongruent flavour combinations are generally both semantically and perceptually dissimilar, flavour combinations that are perceptually dissimilar but semantically congruent are less common. The semantically congruent combinations here comprised five perceptually similar pairs (e.g., watermelon and mango) and five perceptually dissimilar pairs (e.g., chocolate and coffee).

#### 2.3.3 Part 1, procedure

In the first part of the flavour battery, 25 trials were administered comprising the set of 20 flavour combination pairs plus five trials in which each member of the pair was the same. This small number of ‘same’ trials was used in order to reduce any effect from satiety, as satiety can significantly modify gustatory processing (Small et al., 2001). Accordingly the battery comprised 20 ‘different’ and five ‘same’ flavour pairs. Trials were presented in randomised order. Jelly beans were placed in the subject's hand out of vision by the examiner, and the subject was instructed to lift them directly to the mouth, to minimise any use of colour cues. The subject rinsed their mouth with water between jelly beans in each pair. While tasting the second flavour in the pair for each trial, the subject was asked to make three decisions relating respectively to the perceptual, congruence and identification components of the battery (see Table 2). Practice trials were given initially to ensure that the subject understood each task; no feedback was given about performance during the test. The subject rinsed again between each flavour pair.

#### 2.3.4 Part 1, task 1: flavour perception

To assess flavour perception, on each trial the subject was asked first to decide if the two flavours in the pair were the same or different. Performance on this perceptual test was expressed as a value of A-prime for detection of ‘same’ (‘hit’) trials (A-prime is a non-parametric analogue of the d-prime used to assess hit rate for detection of relatively infrequent ‘hit’ events versus the false alarm rate, over small numbers of trials: Grier, 1971).

#### 2.3.5 Part 1, task 2: congruence of flavour combinations

To assess subjects' judgment of flavour combination congruence, on each ‘different’ trial the subject was then asked to decide if the flavour combination in that pair was a usual or unusual flavour combination (i.e., if the combination was semantically congruent or incongruent). To avoid contamination by trials on which the subject erroneously classified non-identical flavours as ‘same’, such error trials were not included in the flavour congruence analysis and the subject's proportional score was calculated accordingly.

#### 2.3.6 Part 1, task 3: flavour identification

To assess flavour identification, while tasting the second flavour in the pair the subject was presented with a set of three word-picture combinations representing the target flavour plus two foils, and asked to choose the word-picture combination matching the target flavour. Foils on each trial were intended to comprise a semantically related and a semantically unrelated flavour item (e.g., the target ‘coffee’ was presented with ‘chocolate’ [related foil] and ‘watermelon’ [unrelated]). This cross-modal matching procedure was repeated on consecutive trials until all twenty target flavours had been presented.

The structure of the first part of the flavour battery is schematised in Table 2.

#### 2.3.7 Part 2: pleasantness of flavour combinations

We reasoned that presenting pairs of flavours simultaneously rather than sequentially should provide information about the affective valence (pleasantness) of different flavour combinations. This affective evaluation might in principle dissociate from the ‘cognitive’ labelling of the combinations as usual or unusual, as assessed using sequential presentation of flavour combinations in the first part of the battery. Accordingly, in the second part of the flavour battery, the subject was again presented with the set of 20 ‘different’ flavour combinations in randomised order but the jelly beans in each pair were tasted simultaneously rather than sequentially. For each trial the subject was asked to classify the flavour combination as pleasant, neutral or unpleasant.

## 3 Results

### 3.1 Odour processing

On the UPSIT Cases 2, 3 and 4 performed below the 5th percentile for age and gender; patient 1 (with SD) performed within normal limits for his age and gender. All healthy control subjects performed within normal limits for their age and gender (Table 3).

### 3.2 Flavour perception

On the perceptual component of the first part of the novel flavour battery, the three patients with SD performed equivalently to healthy control subjects (A-prime ≥ .9) (Table 3). In contrast the performance of Case 4 (with LPA) was clearly inferior (A-prime .73). To address the possibility this was simply the result of a working memory deficit, the test was readministered to Case 4 presenting the jelly beans in each pair simultaneously rather than sequentially. Even under this condition (which removed any working memory demand), the performance of Case 4 remained inferior (A-prime .79).

### 3.3 Flavour combination congruence

On the determination of congruence of flavour combinations the patient group performed significantly worse than healthy controls (*p* < .05, Mann Whitney *U*). No patient's performance was meaningfully different from chance, whereas all healthy control subjects were able to perform this task (proportional scores ≥ 74%, based on the intended congruence classification) (Table 3). Neither in patients nor controls was performance on this test correlated with a measure of non-verbal, fluid intelligence (Raven's progressive matrices) dependent on executive function. Examining the errors made by patients on the flavour congruence task as a group, misidentification of semantically congruent flavour pairs as incongruent was as likely to occur for perceptually similar pairs (8 errors) as for semantically congruent but perceptually dissimilar pairs (7 errors).

### 3.4 Flavour identification

On the flavour identification task patients as a group performed significantly worse than healthy controls (*p* < .05, Mann Whitney *U*). However, Cases 1 (early SD) and 4 (LPA) performed at the lower boundary of the healthy control range (Table 3), whereas Cases 2 and 3 (moderately severe SD) performed near chance on this test. Flavour identification performance was positively correlated with odour identification performance assessed using the UPSIT both in patients (*r* = .81; *p* = .09) and healthy controls (*r* = .79; *p* < .05). Performance on the flavour identification task also positively correlated with performance on the flavour congruence task in both patients (*r* = .9; *p* = .05) and controls (*r* = .77; *p* < .05). Examining the errors made by patients on the flavour identification task as a group, target flavours were more often confused with semantically related foils (20/34 or 59% of all errors) than with unrelated foils (14/34 or 41% of all errors); this disproportion was not statistically significant [*p* > .1, *χ*^2^(1)], perhaps due to the relatively small number of trials. Within the set of semantically related errors, perceptually similar errors (10) and perceptually dissimilar errors (10) were equally frequent.

### 3.5 Pleasantness of flavour combinations

In evaluating subjects' classifications of the pleasantness of flavour combinations on the second part of the flavour battery, we assumed that combinations classified as ‘pleasant’ would be more likely to correspond to standard (congruent) rather than non-standard (incongruent), while combinations classified as ‘unpleasant’ would more likely correspond to non-standard (incongruent) rather than standard (congruent). This assumption is not uncommonly violated in everyday life (indeed, such violations are the basis for much avant-garde cuisine): in the specific context of this experiment, the data for control subjects provided a built-in check on the limitations of the assumption with respect to normal individuals (Table 3). We analysed subjects' responses to assess how far pleasantness classification was in agreement with the intended congruence classification; combinations classified as ‘neutral’ were excluded from the analysis as indeterminate, and the subject's proportional score was adjusted accordingly (Table 3; Case 3 did not complete this test). In contrast to their performance on the flavour congruence task, the pleasantness classification scores of Cases 1 (early SD) and 2 (moderately severe SD) overlapped with the healthy control range. The performance of Case 4 (LPA) on the flavour congruence and affective tasks was similar.

## 4 Discussion

The evidence we have presented here suggests that patients with SD have impaired processing of flavour information. Whereas perceptual discrimination of flavours was comparable to healthy control subjects, identification of flavours and determination of the congruence of flavour combinations was deficient. This pattern would be consistent with disordered semantic processing of flavours (an associative agnosia). Taken together with previous evidence for analogous semantic defects in other sensory modalities (Hodges and Patterson, 2007; Bozeat et al., 2000; Coccia et al., 2004; Rami et al., 2007; Luzzi et al., 2007), the present evidence for a deficit of flavour knowledge in SD supports the hypothesis that the semantic deficit in SD is truly ‘pan-modal’. In contrast, we found evidence for a different pattern of deficits in a case of LPA (a syndrome of focal degeneration involving peri-Sylvian cortical areas that is both clinically and anatomically distinct from SD): this patient had impaired perceptual discrimination of flavours, relatively preserved identification of flavours and impaired ability to assess the congruence of flavour combinations. This pattern would be consistent with a primary perceptual defect of flavour processing.

Our findings suggest a mechanism for the development of food fads and other abnormal eating behaviours in SD and other degenerative disorders. The findings further suggest that particular abnormalities of eating behaviour in different disorders may be underpinned by distinct neuropsychological deficits. Degraded semantic associations and reduced ability to assess the ‘appropriateness’ of particular foods and food combinations might lead to food faddism and unusual food preferences in SD: this may involve a loss of item-specific or contextual knowledge about particular foods and food combinations due to an inability to integrate information from different modalities contributing to flavour knowledge (e.g., raw carrot is eaten, while raw potato generally is not; pepper and strawberry are individually standard flavourings, while their combination is highly unusual). Impaired cortical perceptual analysis might lead to distorted or unpleasant flavour percepts in other conditions, such as LPA. However, it is noteworthy that abnormalities of eating behaviour were clinically significant in only two of the patients in this series, suggesting that additional factors modulate the development of such behaviours: one such factor may be disease severity, since neuropsychological deficits commonly predate the onset of clinical symptoms. Although the sample size was small and therefore any correlation analysis should be interpreted with caution, odour and flavour identification performance was correlated for both patients and controls in our series. This is consistent with previous evidence that both olfactory and gustatory cues contribute importantly to flavour processing (Rolls, 2005; Small et al., 2004), and with evidence for specific olfactory and gustatory deficits in patients with TL degenerations (Mendez and Ghajarnia, 2001; Broggio et al., 2001; Gorno-Tempini et al., 2004; Rami et al., 2007; Luzzi et al., 2007).

Identification of flavours and judgments about the congruence of flavour combinations may tap different aspects of flavour knowledge. It is likely that performance on the flavour congruence task here was influenced by executive and perceptual as well as by purely semantic processes. However, we argue that these other factors are unlikely to account entirely for the consistent difficulty that patients exhibited on this task. Performance was not correlated (in this very small sample) with a standard general measure of non-verbal intelligence (Raven's progressive matrices) that incorporates executive processing. The overall pattern of errors on the flavour battery suggests that semantically similar flavours were more likely to be confused than semantically dissimilar flavours, while perceptual similarity did not affect patients' accuracy for cross-modal matching of flavours or determination of flavour congruence. As foods are typically consumed in combinations that are learned over the course of a lifetime's exposure to the mores of an individual's culture, we would argue that flavour congruence is a sensitive probe of higher order semantic knowledge about flavours. A caveat to this is suggested by the evidence of Case 4 here: this patient is likely to have a cortical perceptual deficit but also performed poorly on the flavour congruence task. We interpret this as evidence that semantic processing of flavours depends on accurate perceptual coding.

A further clue to the nature of the flavour processing deficit may lie with patients' performance assessing the pleasantness of flavour combinations (Table 3). The performance of patients with SD on the flavour congruence task (intended as a cognitive decision based on knowledge of food combinations) and the pleasantness task (intended as an affective decision based on the hedonic value of flavour combinations) was dissociable, suggesting that these tasks may tap different kinds of flavour processing. It is possible that pleasantness judgments about flavour combinations here were driven by individual flavours in the combinations (since the ‘incongruent’ combinations contained a higher proportion of flavours, such as licorice, which might themselves more frequently elicit ‘unpleasant’ judgments). Ideally, the pleasantness ratings would also have been obtained for the individual flavours. However, even if pleasantness judgments were in fact driven mainly by individual flavours, the data suggest a dissociation of affective versus cognitive processing of flavour information: inspecting Table 3, Case 2 was near chance on the individual flavour identification task but showed a pleasantness rating comparable to controls, while conversely, Case 4 fell well outside the control range on the pleasantness rating but approached the control range for individual flavour identification. Whereas patients with SD may have been able to use pleasantness to compensate for impaired flavour identification (perhaps by accessing parallel limbic information about the quality of their experience), this was not the case for the patient with LPA (presumably because the flavour percept itself was degraded). We speculate that these findings might signify a chemosensory analogue to other kinds of complex stimuli such as faces, for which both partial dissociations and interactions between perceptual, affective and semantic (identification) mechanisms are well documented (Calder and Young, 2005). However, further work with larger cohorts and closer control of individual flavour semantic versus affective properties is required to settle this issue.

There are several caveats to the present study. Neuropsychological procedures based on serial comparisons of stimuli are of uncertain ecological validity, and this may apply especially to flavours. The use of candies is likely to have modified the task and introduced a greater requirement for executive control (since in a basic sense, most of the stimuli were ‘sweet’). Documenting a flavour defect as here does not resolve the relative contributions of olfactory, gustatory and other flavour components. Moreover, the subject numbers in this study were small. Allowing for these caveats, while we cannot draw firm conclusions about the brain basis for the flavour processing deficits we have identified here, our findings are consistent with emerging evidence for a hierarchical organisation of flavour processing and the brain networks that mediate this in the healthy brain. It is likely that the breakdown of flavour knowledge and other aspects of abnormal eating behaviour in SD result from damage involving the temporal poles and OFC (Whitwell et al., 2007). These areas are sites of higher-order gustatory and olfactory association cortex, which has been implicated in various aspects of flavour integration and semantic processing in normal subjects (Rolls, 2005; Small et al., 2004). These processes may be at least partly separable from processes involved in representing the perceptual and hedonic characteristics of flavours (Small et al., 2001; De Araujo et al., 2003; Hinton et al., 2004), though these aspects are very likely to interact under natural eating conditions. In contrast, a primary cortical perceptual deficit would be predicted where (as in Case 4 here) the pathological process involves peri-Sylvian areas including the insula. These regions contain early gustatory and olfactory cortices that represent elementary features of the flavour percept (Rolls, 2005; De Araujo et al., 2003). Profiles of cortical chemosensory dysfunction may also associate with tissue pathology for diseases in the neurodegenerative spectrum: Luzzi et al. (2007) reported a predominantly perceptual defect of olfactory processing in patients with Alzheimer's disease, which is likely to be the histopathological substrate in a high proportion of cases of LPA (Gorno-Tempini et al., 2008). These hypotheses suggest clear directions for future work.

## Figures and Tables

**Table 1:** Summary of patient characteristics, background neuropsychology and MR findings.

Patient	Age/gender	Clinical diagnosis	Duration of clinical illness	Abnormal eating behaviour	Background neuropsychology													Brain MRI findings
					Raven's Matrices^a^		Camden Memory^b^		Rep^c^ /30	Picture naming^c^		Synonyms (concrete)^d^		Famous faces^e^		Object decision^f^		
					raw/12	% ile	raw/30	% ile		raw/20	% ile	raw/25	% ile	raw/12	% ile	raw/20	% ile	
1	55 M	SD	4 y	–	8	50–75	30	50–100	30	5	<5	16	5–10	9	25	20	90–100	Antero-inferior L > R TL atrophy
2	59 M	SD	7 y	Preference for foods (pineapple, licorice) previously disliked	8	75–90	29	25–50	30	0	<5	14	1–5	0	<5	15	10–20	Antero-inferior L > R TL atrophy, inferior FL atrophy
3	63 F	SD	7 y	–	5	50	14	<1	30	0	<5	15	5	2	<5	8	<1	Antero-inferior L > R TL atrophy, inferior FL atrophy
4	56 M	LPA	3 y	Sweet tooth, red wine ‘bitter’, honey unpleasant	1	<5	24	<1	30	12	<5	19	25	10	50	18	50–75	L > R TL, peri-Sylvian atrophy

Key: F, female; FL, frontal lobe; L, left; M, male; R, right; Rep, polysyllabic word repetition.

a Warrington EK. Ravens Advanced Progressive Matrices, Recognition memory test: manual. California, USA: Western Psychological Services, 1984.

b Warrington EK. The Camden memory tests: manual. East Sussex, UK: Psychology Press, 1996.

c Polysyllabic word repetition and easy picture naming tests; percentiles calculated from previous healthy control sample (*n* = 42; details available from the authors).

d Warrington EK et al. Single word comprehension: a concrete and abstract word synonym test. Neuropsychological Rehabilitation 1998; 8: 143–154.

e Warrington EK. James, M. An experimental study of facial recognition in patients with unilateral cerebral lesions. Cortex 1967; 3: 317–326.

f Warrington EK, James M. The visual object and space perception battery. Bury St. Edmunds: Thames Valley Test Company, 1991.

**Table 2:** Structure of the flavour battery (first part).

Pair No.^a^	Flavour pair		**Task 1**: Same or different flavours?	**Task 2**: Usual (congruent) or unusual (incongruent) combination?	**Task 3**: Which flavour is it?		
Jelly bean 1	**Jelly bean 2**	Flavour identification: word/picture match^b^		
Close foil	Distant foil	Target
1	Pickle	**Pickle**	S	–	Pepper	Mango	Pickle
2	Coconut	**Coconut**	S	–	Vanilla	Pepper	Coconut
3	Peppermint	**Peppermint**	S	–	Chocolate	Pear	Peppermint
4	Chocolate	**Chocolate**	S	–	Coffee	Watermelon	Chocolate
5	Licorice	**Licorice**	S	–	Chocolate	Lemon	Licorice
6	Chocolate	**Peanut butter**	D	C	Chocolate	Licorice	Peanut butter
7	Chocolate	**Coffee**	D	C	Chocolate	Watermelon	Coffee
8	Vanilla	**Strawberry**	D	C	Black currant	Pickle	Strawberry
9	Vanilla	**Raspberry**	D	C	Strawberry	Pepper	Raspberry
10	Cinnamon	**Apple**	D	C	Pear	Licorice	Apple
11	Orange	**Lemon**	D	C	Orange	Toffee	Lemon
12	Strawberry	**Black currant**	D	C	Strawberry	Licorice	Black currant
13	Watermelon	**Mango**	D	C	Watermelon	Chocolate	Mango
14	Mango	**Orange**	D	C	Mango	Peanut butter	Orange
15	Apple	**Pear**	D	C	Apple	Peppermint	Pear
16	Coffee	**Watermelon**	D	IC	Mango	Peanut butter	Watermelon
17	Watermelon	**Cinnamon**	D	IC	Apple	Strawberry	Cinnamon
18	Strawberry	**Pepper**	D	IC	Pickle	Coffee	Pepper
19	Lemon	**Toffee**	D	IC	Vanilla	Pickle	Toffee
20	Pickle	**Vanilla**	D	IC	Strawberry	Watermelon	Vanilla
21	Pear	**Peppermint**	D	IC	–	–	–
22	Mango	**Pickle**	D	IC	–	–	–
23	Pepper	**Coconut**	D	IC	–	–	–
24	Licorice	**Apple**	D	IC	–	–	–
25	Peanut butter	**Licorice**	D	IC	–	–	–

Key: C, congruent (intended classification); D, different; IC, incongruent (intended classification); S, same. Identification task based on jelly bean 2 in each pair (shown in bold).

a Pairs presented in randomised order.

b Screen positions of target and foils randomised.

**Table 3:** Summary of subject performance on olfactory and flavour tests.

Subject age, gender	Olfactory (UPSIT)^a^		Flavours			
Part 1			Part 2
Patients	Raw/40	%ile	Task 1: Perception (A-prime)	Task 2: Flavour combination congruence^d^ (proportion correct)	Task 3: Flavour identificationa,c (proportion correct)	Flavour combination pleasantness^d^ (proportion in agreement with intended congruence classification)^e^
1 55 M	30	>20	.97	.56	.75	.76
2 59 M	17	<5	.9	.47	.45	.81
3 63F	15	<5	.91	.33	.4	N/A
4 56 M	19	<5	.73 (.79)^b^	.59	.7	.53

Healthy controls						
1 67 M	34	>50	1	.85	.9	.74
2 52 M	28	>10	.95	.75	.75	.66
3 62F	31	>15	.93	.89	.9	.73
4 60 M	35	>50	1	.9	.9	.81
5 65 M	33	>40	.99	.74	.85	.73
6 63F	30	>10	.93	.84	.85	.76

a Presented in cross-modal simultaneous word–picture matching format.

b Simultaneous presentation of jelly beans in each pair.

c Chance performance = .33.

d Chance performance = .5.

e See text for explanation.

## References

[bib1] Bozeat S., Lambon Ralph M.A., Patterson K., Garrard P., Hodges J.R. (2000). Non-verbal semantic impairment in semantic dementia. Neuropsychologia.

[bib2] Broggio E., Pluchon C., Ingrand P., Gil R. (2001). Taste impairment in Alzheimer's disease. Revue Neurologique (Paris).

[bib3] Calder A.J., Young A.W. (2005). Understanding the recognition of facial identity and facial expression. Nature Reviews Neuroscience.

[bib4] Coccia M., Bartolini M., Luzzi S., Provinciali L., Lambon Ralph M.A. (2004). Semantic memory is an amodal, dynamic system: Evidence from the interaction of naming and object use in semantic dementia. Cognitive Neuropsychology.

[bib6] De Araujo I.E.T., Rolls E.T., Kringelbach M.L., McGlone F., Phillips N. (2003). Taste-olfactory convergence and the representation of the pleasantness of flavour in the human brain. European Journal of Neuroscience.

[bib7] Desgranges B., Matuszewski V., Piolino P., Chételat G., Mézenge F., Landeau B. (2007). Anatomical and functional alterations in semantic dementia: A voxel-based MRI and PET study. Neurobiology of Aging.

[bib8] Doty R.L., Shaman P., Dann M. (1984 March). Development of the university of Pennsylvania smell identification test: A standardized microencapsulated test of olfactory function. Physiology and Behavior.

[bib9] Gorno-Tempini M.L., Brambati S.M., Ginex V., Ogar J., Dronkers N.F., Marcone A. (2008). The logopenic/phonological variant of primary progressive aphasia. Neurology.

[bib10] Gorno-Tempini M.L., Rankin K.P., Woolley J.D., Rosen H.J., Phengrasamy L., Miller B.L. (2004). Cognitive and behavioral profile in a case of right anterior temporal lobe neurodegeneration. Cortex.

[bib11] Grier J.B. (1971). Nonparametric indexes for sensitivity and bias: Computing formulas. Psychological Bulletin.

[bib12] Hinton E.C., Parkinson J.A., Holland A.J., Arana F.S., Roberts A.C., Owen A.M. (2004). Neural contributions to the motivational control of appetite in humans. European Journal of Neuroscience.

[bib13] Hodges J.R., Patterson K. (2007). Semantic dementia: A unique clinicopathological syndrome. Lancet Neurology.

[bib14] Kareken D.A., Mosnik D.M., Doty R.L., Dzemidzic M., Hutchins G.D. (2003). Functional anatomy of human odour sensation, discrimination, and identification in health and aging. Neuropsychology.

[bib15] Lambon Ralph M.A., Patterson K. (2008). Generalization and differentiation in semantic memory: Insights from semantic dementia. Annals of the New York Academy of Sciences.

[bib16] Luzzi S., Snowden J.S., Neary D., Coccia M., Provinciali L., Lambon Ralph M.A. (2007). Distinct patterns of olfactory impairment in Alzheimer's disease, semantic dementia, frontotemporal dementia, and corticobasal degeneration. Neuropsychologia.

[bib17] Mendez M.F., Ghajarnia M. (2001). Agnosia for familiar faces and odours in a patient with right temporal lobe dysfunction. Neurology.

[bib18] Neary D., Snowden J.S., Gustafson L., Passant U., Stuss D., Black S. (1998). Frontotemporal lobar degeneration: A consensus on clinical diagnostic criteria. Neurology.

[bib19] Rami L., Loy C.T., Hailstone J., Warren J.D. (2007). Odour identification in frontotemporal lobar degeneration. Journal of Neurology.

[bib20] Rolls E.T. (2005). Taste, olfactory and food texture processing in the brain, and the control of food intake. Physiology and Behaviour.

[bib21] Rosen H.J., Allison S.C., Ogar J.M., Amici S., Rose K., Dronkers N. (2006). Behavioral features in semantic dementia versus other forms of progressive aphasias. Neurology.

[bib22] Royet J.P., Koening O., Gregoire M.C., Cinotti L., Lavenne F., Le Bars D. (1999). Functional anatomy of perceptual and semantic processing for odours. Journal of Cognitive Neuroscience.

[bib23] Savic I. (2002). Brain imaging studies of the functional organization of human olfaction. Neuroscientist.

[bib24] Small D.M., Voss J., Mak E., Simmons K.B., Parrish T., Gitelman D. (2004). Experience-dependent neural integration of taste and smell in the human brain. Journal of Neurophysiology.

[bib25] Small D.M., Zatorre R.J., Dagher A., Evans A.C., Jones-Gotman M. (2001). Changes in brain activity related to eating chocolate: From pleasure to aversion. Brain.

[bib26] Snowden J.S., Bathgate D., Varma A., Blackshaw A., Gibbons Z.C., Neary D. (2001). Distinct behavioural profiles in frontotemporal dementia and semantic dementia. Journal of Neurology, Neurosurgery and Psychiatry.

[bib27] Whitwell J.L., Sampson E.L., Loy C.T., Warren J.E., Rossor M.N., Fox N.C. (2007). VBM signatures of abnormal eating behaviours in frontotemporal lobar degeneration. NeuroImage.

